# The Faraday efficiency of nitrate reduction reactions and selectivity of final product for electrocatalysts Cu/Co-OH/CF by tuning the content of Co source

**DOI:** 10.3389/fchem.2025.1599613

**Published:** 2025-07-18

**Authors:** Li Li, Qiang Wang, Yanling Chen, Xuan Chen, Quguo Shi

**Affiliations:** ^1^ College of Mechanical and Electrical Engineering, Suzhou University, Suzhou, Anhui, China; ^2^ Key Laboratory of Spin Electron and Nanomaterials of Anhui Higher Education Institutes, Suzhou University, Suzhou, Anhui, China

**Keywords:** nitrate reduction reaction, electrocatalysts, Cu/Co-OH/CF, selectivity, copper foam

## Abstract

Efficient and selective electrocatalytic reduction of nitrate (NO_3_^−^) to ammonia (NH_3_) is a crucial step in addressing environmental and energy challenges. Here, we present the synthesis and characterization of Cu/CF, Co-OH/CF, and a series of Cu/Co-OH/CF electrocatalysts on copper foam (CF) substrates using a hydrothermal method. These catalysts were utilized as working electrodes for nitrate reduction, resulting in the production of NH_3_. Through X-ray photoelectron spectroscopy (XPS) analysis, we confirm the presence of metallic Cu species and oxidized Co^2+^ states in the electrocatalysts, indicating the successful formation of Cu/Co-OH/CF composites. By precisely controlling the quantities of cobalt in the composite, we demonstrate the ability to finely tune the nitrate reduction efficiency and selectivity of the final product. Notably, our findings reveal that the Co-(OH) species in the Cu/Co-OH/CF electrocatalysts play a pivotal role in determining the selectivity of the final product by effectively suppressing the undesired hydrogen evolution reaction. Simultaneously, Cu acts as an active component in the reduction of NO_3_^−^ into ammonia. Our work offers valuable guidance for designing advanced electrocatalytic systems with enhanced Faraday efficiency and selectivity, thereby contributing to the development of sustainable and efficient nitrate reduction technologies.

## 1 Introduction

Ammonia (NH_3_) is an indispensable chemical compound with diverse industrial applications, serving as a pivotal fuel source, a vital component in fertilizers, and a highly effective gas reducing agent (Wang et al., 2021; Foster et al., 2018; Gruber and Galloway, 2008; Wang et al., 2018; Suryanto et al., 2019). The current predominant method utilized for ammonia synthesis is the renowned Haber-Bosch process, which involves the reaction N_2_+3H_2_ → 2NH_3_ and necessitates operating under high temperatures and pressures (Giddey et al., 2013; Jiao and Xu, 2019; Wu et al., 2021; Kyriakou et al., 2017; Jiang et al., 2025a). However, these rigorous reaction conditions lead to substantial energy consumption, thereby rendering the process inherently unsustainable. In comparison, the electrochemical nitrate reduction (NRR) route presents a compelling alternative that holds great promise (Song et al., 2023; Xiong et al., 2024; Mu et al., 2024; Jiang et al., 2025b). Notably, this approach offers a multitude of advantages, including the utilization of abundant resources, cost-effectiveness, and remarkably low energy demand (Gan et al., 2024; Liang et al., 2023; Ahmed et al., 2024). By harnessing the power of electrochemistry, the NRR method presents an innovative pathway for ammonia production that aligns with the principles of sustainability and efficiency.

However, the electrochemical NRR process is a highly intricate and multifaceted reaction, involving a complex series of eight electron transfer processes. This intricacy not only renders the NRR process susceptible to the formation of potentially toxic by-products like nitrite (NO_2_^−^) (Hu et al., 2021; Li et al., 2021; Chen et al., 2023), but it also introduces competition with the hydrogen evolution reaction (HER) (Hirakawa et al., 2017; Jin et al., 2023; Chebrolu et al., 2023). As a result, the primary challenge in selecting and designing NRR catalysts lies in effectively enhancing the NRR activity while concurrently minimizing side reactions and competitive processes. By addressing this challenge, researchers aim to optimize the yield and Faraday efficiency of ammonia, thereby establishing a pathway towards sustainable and efficient ammonia production through electrochemical means. Consequently, the development of high-performance catalysts capable of promoting selective NRR and suppressing unwanted reactions is of paramount importance in advancing the field of electrochemical ammonia synthesis.

Among the various metal catalysts explored for electrochemical NRR, heterogeneous catalysts based on copper (Cu) have emerged as exceptional candidates, exhibiting remarkable Faraday efficiency exceeding 80% even at low reduction potentials (Wang et al., 2020; Bae et al., 2007; Butcher and Gewirth, 2016; Comisso et al., 2012). This outstanding performance underscores the immense promise of Cu-based catalysts in facilitating NRR. Extensive research conducted on bulk Cu catalysts has revealed a crucial phenomenon known as crystal plane selection effect during the electrocatalytic nitrate reduction reaction (Pérez-Gallent et al., 2017; Bae and Gewirth, 2009; Ullah et al., 2023). These findings emphasize that the size and structure of Cu species play a pivotal role in determining the catalyst’s activity and selectivity (Chen et al., 2025). However, it is imperative to note that Cu alone does not adequately suppress the undesired HER, necessitating the exploration of innovative strategies and alternative approaches. By developing synergistic methodologies and exploring novel catalyst compositions, researchers aim to overcome the limitations of Cu-based catalysts and enhance their selectivity towards NRR, thereby opening new avenues for efficient and sustainable ammonia synthesis.

In this work, we conducted a systematic investigation on the fabrication of different electrocatalysts, including Cu/CF, Co(OH)_2_/CF, and Cu/Co(OH)_2_/CF, employing a facile hydrothermal method. These electrocatalysts were specifically designed on copper foam or mesh (CF) substrates and served as the reduction working electrodes for the nitrate (NO_3_^−^) reduction process, with the ultimate goal of producing ammonia. Our findings revealed a remarkable control over the NRR efficiency and selectivity of the final product by manipulating the quantities of Co incorporated into the composite catalysts. Through careful tuning of the Co content, we were able to achieve enhanced NRR performance and fine-tune the selectivity towards ammonia formation. This simple and low-cost strategy presents a significant breakthrough in resolving the selectivity challenges commonly encountered in NRR reactions. By providing an effective means to modulate the catalyst composition, our work contributes to the development of scalable and cost-efficient approaches for achieving high selectivity in NRR. This research lays the foundation for further advancements in electrocatalysis and offers valuable insights into the design and optimization of catalyst materials for sustainable and selective ammonia synthesis.

## 2 Results and discussions

**FIGURE 1:**
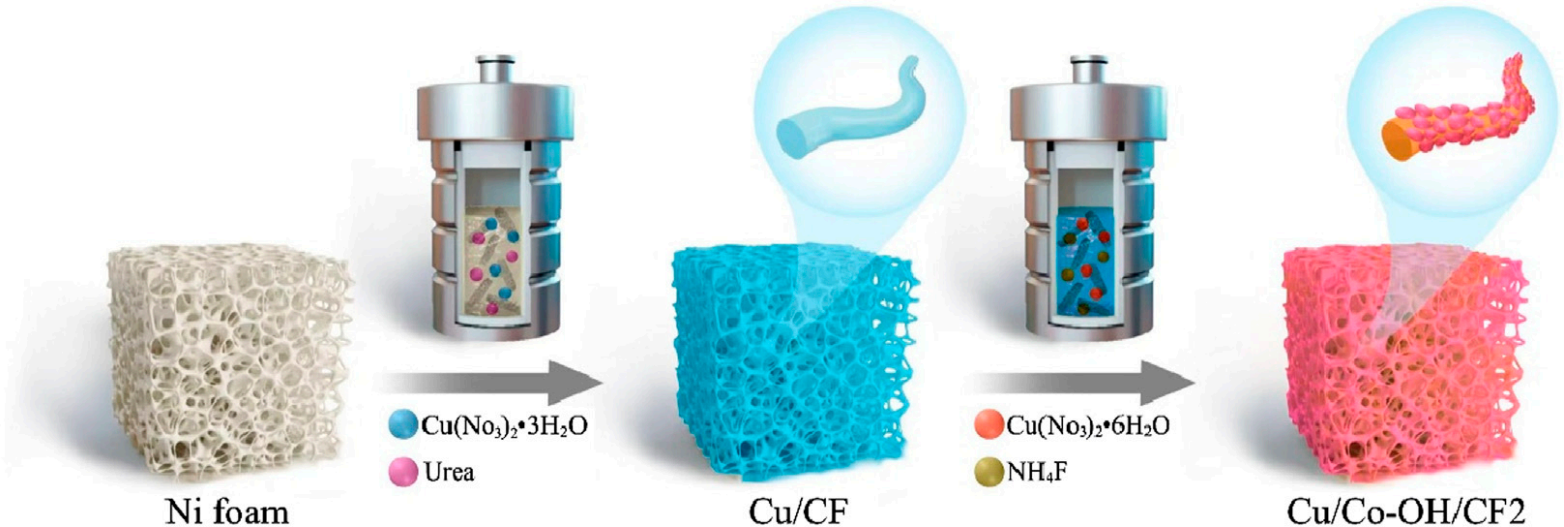
Detailed process flow diagram of the synthetic/preparation route for Cu/Co-OH/CF2.

**FIGURE 2:**
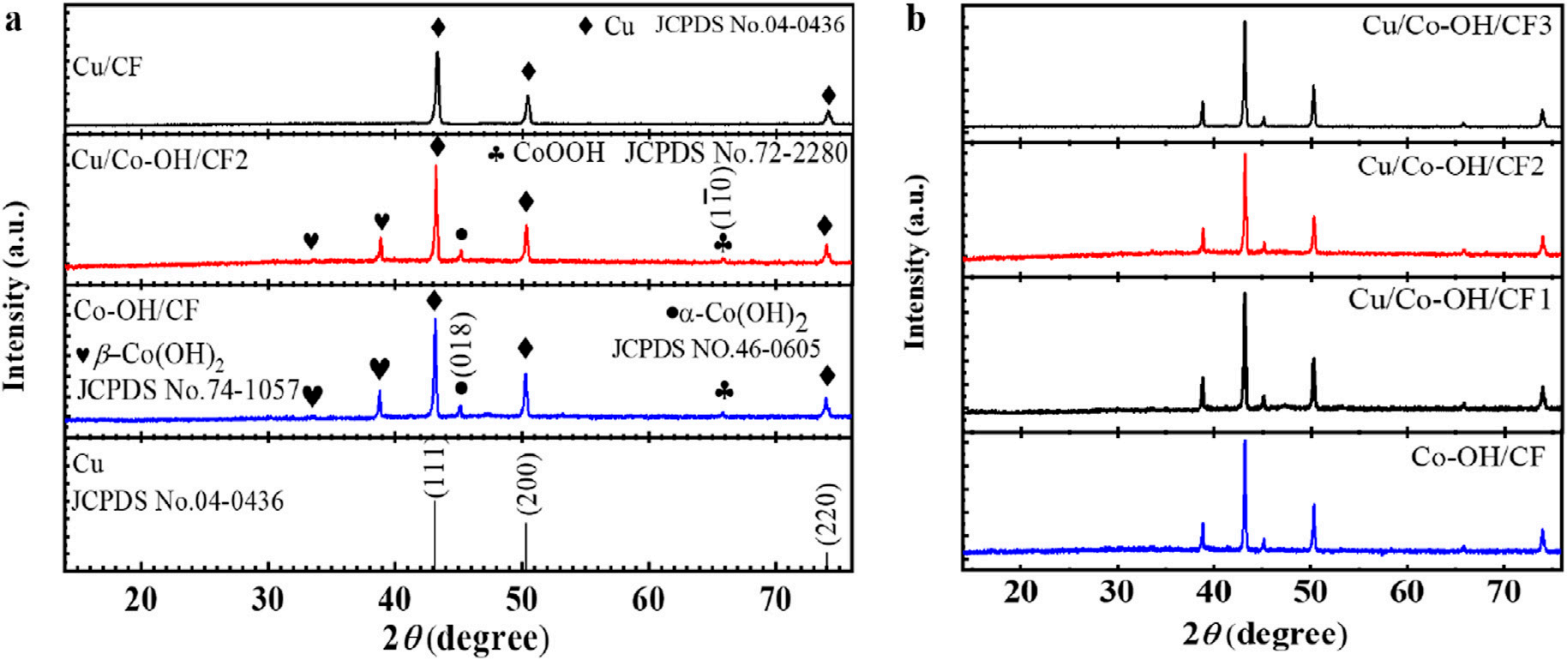
**(a)** XRD patterns of samples Cu/CF, Cu/Co-OH/CF2, Co-OH/CF, and Cu standard card; **(b)** XRD patterns of Cu/Co-OH/CF1-3 and Co-OH/CF.

To clearly illustrate the synthetic/preparation route of Cu/Co-OH/CF2 Figure 1 shows its detailed process flow diagram. Figures 2a,b present the XRD patterns of all the synthesized samples. The XRD pattern of Cu/CF reveals three distinct peaks at 43.3°, 50.4°, and 71.2°, corresponding to the (111), (200), and (220) planes of bulk Cu (JCPDS No. 04-0436), respectively. This indicates that after the hydrothermal reaction, the Cu/CF sample predominantly contains Cu. Conversely, the XRD patterns of the Co-OH/CF, Cu/Co-OH/CF1-3 samples exhibit additional peaks, indicating the presence of new crystalline phases. Upon careful analysis, the new peaks at 33.5° and 38.8° can be attributed to the (100) and (011) crystal planes of *β*-Co(OH)_2_ (JCPDS No. 74-1057), respectively. Furthermore, the peak at 45.1° corresponds to the (018) plane of α-Co(OH)_2_ (JCPDS No. 46-0605), while the peaks at 65.8° are identified as the crystal ( $1\bar{1}0$ ) plane of CoOOH (JCPDS No. 72-2280). The relative intensity of each peak in the XRD patterns reveals that copper is the dominant phase, followed by β-Co(OH)_2_, and finally α-Co(OH)_2_ and CoOOH. It is worth noting that the coexistence of *α*-Co(OH)_2_, *β*-Co(OH)_2_, and CoOOH phases has been commonly reported, as *β*-Co(OH)_2_ is more thermodynamically stable than *α*-Co(OH)_2_ (Peng et al., 2017; Yuan et al., 2023). This observation further confirms the coexistence of copper and cobalt hydroxide species in the samples. For subsequent analysis and discussions, the Cu/Co-OH/CF2 sample was selected as a representative due to its superior performance in terms of nitrate reduction rate and selectivity towards the desired final product. This selection highlights the importance of the specific composition and structure of the Cu/Co-OH/CF2 composite in achieving optimal electrocatalytic properties.

**FIGURE 3:**
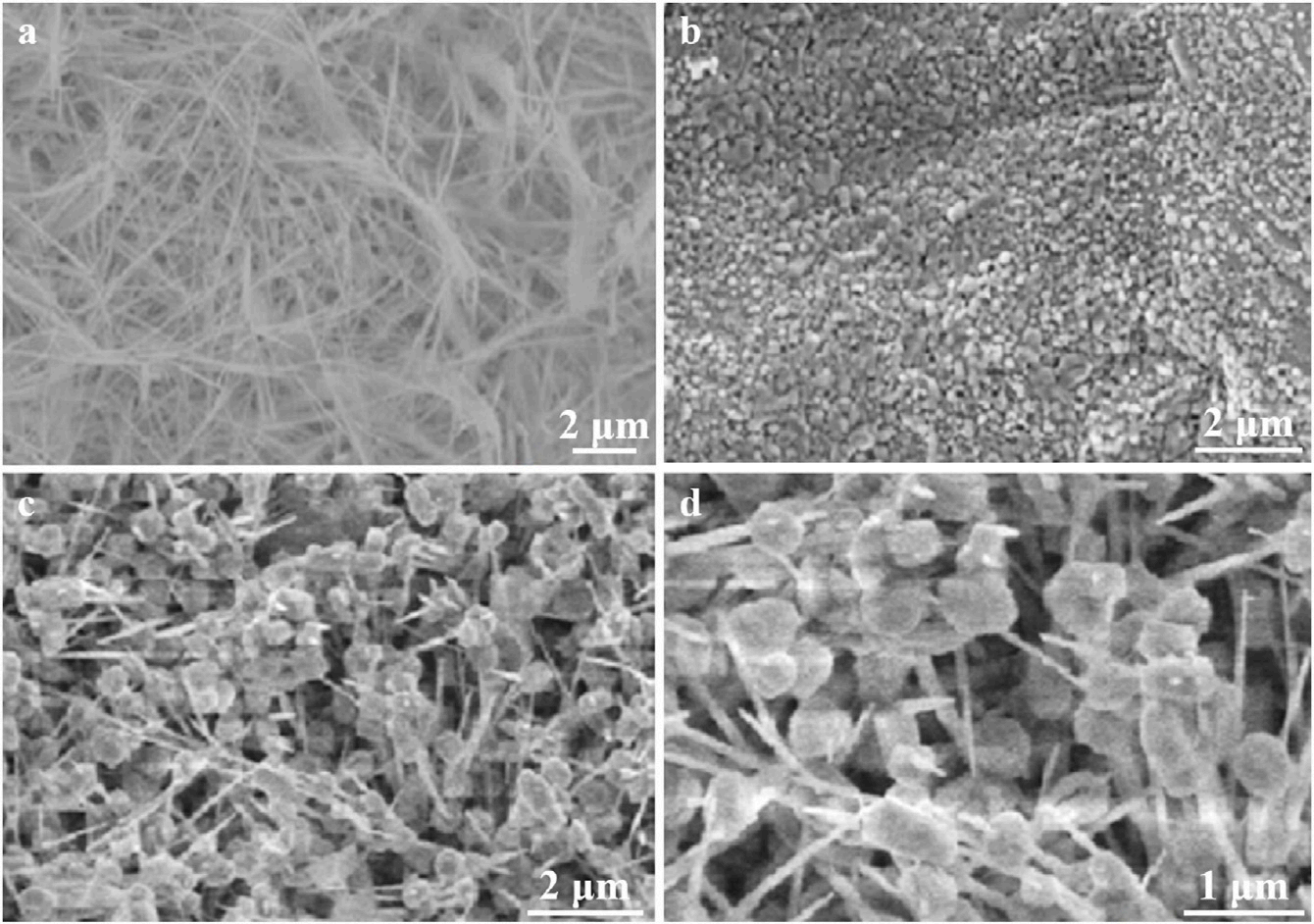
SEM images of sample **(a)** Cu/CF, **(b)** Co-OH/CF, and **(c)** Cu/Co-OH/CF_2_. **(d)** Two times magnification of **(c)**.

Figure 3 presents a comprehensive analysis of the morphological features of the synthesized electrocatalysts: Cu/CF, Co-OH/CF, and Cu/Co-OH/CF2, as revealed through SEM imaging. The Cu/CF sample (Figure 3a) exhibits a distinct network of interconnected copper nanowires, formed through direct growth on the carbon foam substrate. These elongated nanostructures provide an extensive surface area, potentially enhancing electrocatalytic activity by maximizing exposure to reactant species. In contrast, the Co-OH/CF sample (Figure 3b) displays a predominantly spherical morphology, likely originating from the aggregation of initially formed plate-like β-Co(OH)_2_ structures. While spherical structures can offer certain advantages, such as enhanced mass transport, they may also limit the exposure of active sites compared to more open morphologies.

Notably, the Cu/Co-OH/CF2 composite (Figure 3c) showcases a unique hybrid structure characterized by the coexistence of hexagonal nanosheets and intertwined nanowires. This intricate morphology, further emphasized in the magnified image (Figure 3d), is attributed to the synergistic interplay between copper and cobalt species during the synthesis process. The observed hexagonal nanosheets, in particular, are likely facilitated by the incorporation of cobalt hydroxide, suggesting a cooperative effect in the formation of this distinctive architecture. This hybrid structure is anticipated to offer multiple advantages for nitrate reduction. The abundant active sites provided by both nanosheets and nanowires are expected to facilitate efficient charge transfer and mass transport, thereby enhancing catalytic performance. The large interfacial area offered by the nanosheets can promote adsorption and reaction of nitrate ions, while the interconnected nanowire network may facilitate rapid electron transport. Moreover, the hierarchical structure of the composite could potentially enhance electrolyte accessibility and product diffusion. These structural characteristics collectively contribute to the optimization of the Cu/Co-OH/CF2 electrocatalyst for nitrate reduction, potentially leading to improved Faraday efficiency and selectivity.

The morphological differences observed among the three samples highlight the importance of compositional control in tailoring electrocatalyst structures. The formation of nanowires in the Cu/CF sample, nanospheres in the Co-OH/CF sample, and the hybrid structure in the Cu/Co-OH/CF2 composite demonstrate the versatility of the synthesis approach. These findings underscore the potential for further optimization of the catalyst composition and synthesis conditions to achieve desired morphological features and enhance electrocatalytic performance.

**FIGURE 4:**
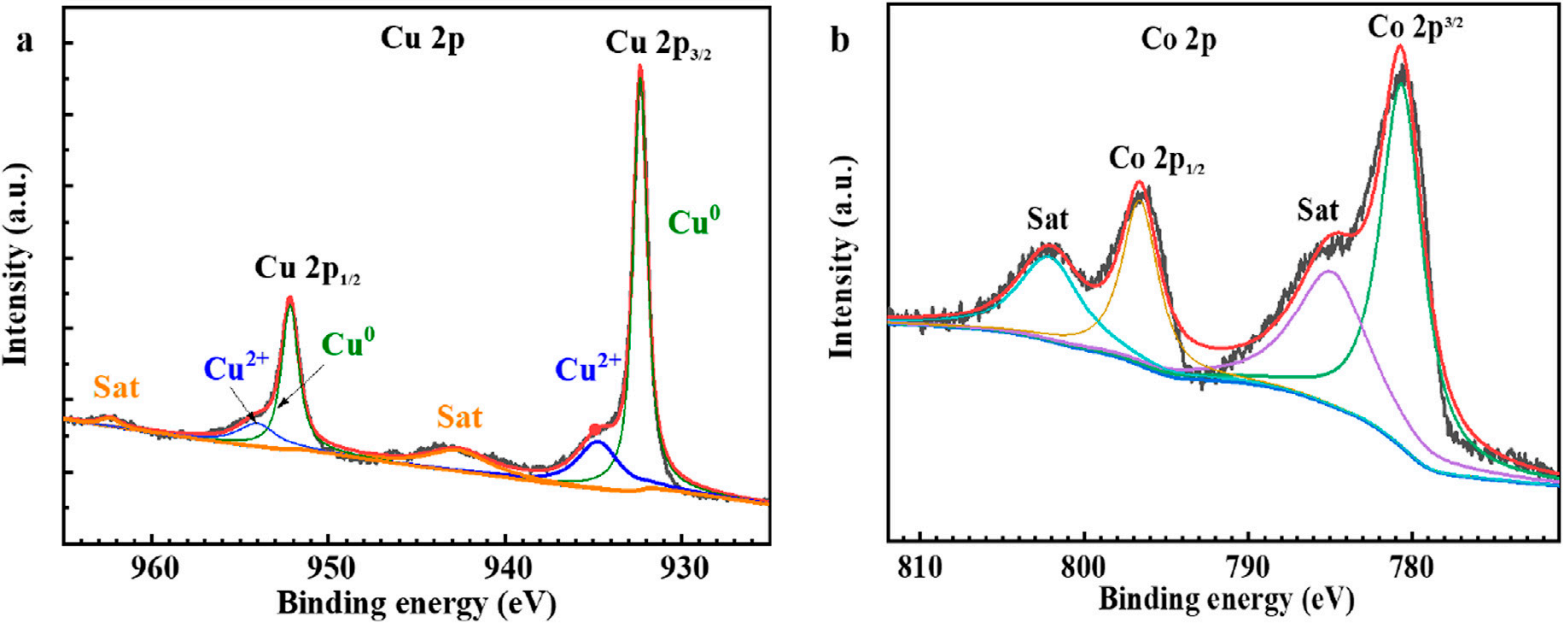
XPS spectra of **(a)** Cu 2p and **(b)** Co 2p of the selected sample Cu/Co-OH/CF2.

To gain further insights into the chemical composition and valence states of the surface of the electrocatalyst samples, X-ray photoelectron spectroscopy (XPS) was employed for characterization. The XPS results of the selected Cu/Co-OH/CF2 sample provide additional evidence regarding the oxidation states of copper and cobalt species, as illustrated in Figures 4a,b respectively.

The analysis of the Cu 2p XPS spectrum reveals that the majority of copper exists in a metallic state, with only a small fraction of Cu^2+^ ions present due to the slight surface oxidation of copper (Figure 4a). This observation is consistent with the literature, which suggests that copper in its pure form tends to exhibit a zero valence state, with minimal surface oxidation (Li et al., 2021; Sun et al., 2024; Zhang et al., 2023). The presence of metallic copper suggests its potential role in facilitating electron transfer during the nitrate reduction process.

In contrast, the XPS spectrum of the Co element indicates the presence of Co^2+^ species. Notably, two prominent peaks at 780.9 eV and 796.7 eV are observed, corresponding to the 2p3/2 and 2p1/2 orbitals of cobalt, respectively. Additionally, accompanying satellite peaks are observed, further confirming the presence of Co^2+^ ions. The positions of these peaks align well with the reported binding energies of either α-Co(OH)_2_ or β-Co(OH)_2_ (Xue et al., 2012; Wang et al., 2014; Liang et al., 2019). The existence of Co^2+^ species indicates the participation of cobalt in the electrocatalytic reactions.

The XPS characterization provides valuable information about the oxidation states of copper and cobalt in the Cu/Co-OH/CF2 sample. The synergistic interplay between these two elements, each in its respective valence state, may contribute to the enhanced Faraday efficiency and selectivity observed in the nitrate reduction process. The metallic copper facilitates electron transfer, while the oxidized cobalt species participate in the catalytic conversion of nitrate. This interplay could optimize the electronic structure of the catalyst and improve its overall performance in nitrate reduction.

**FIGURE 5:**
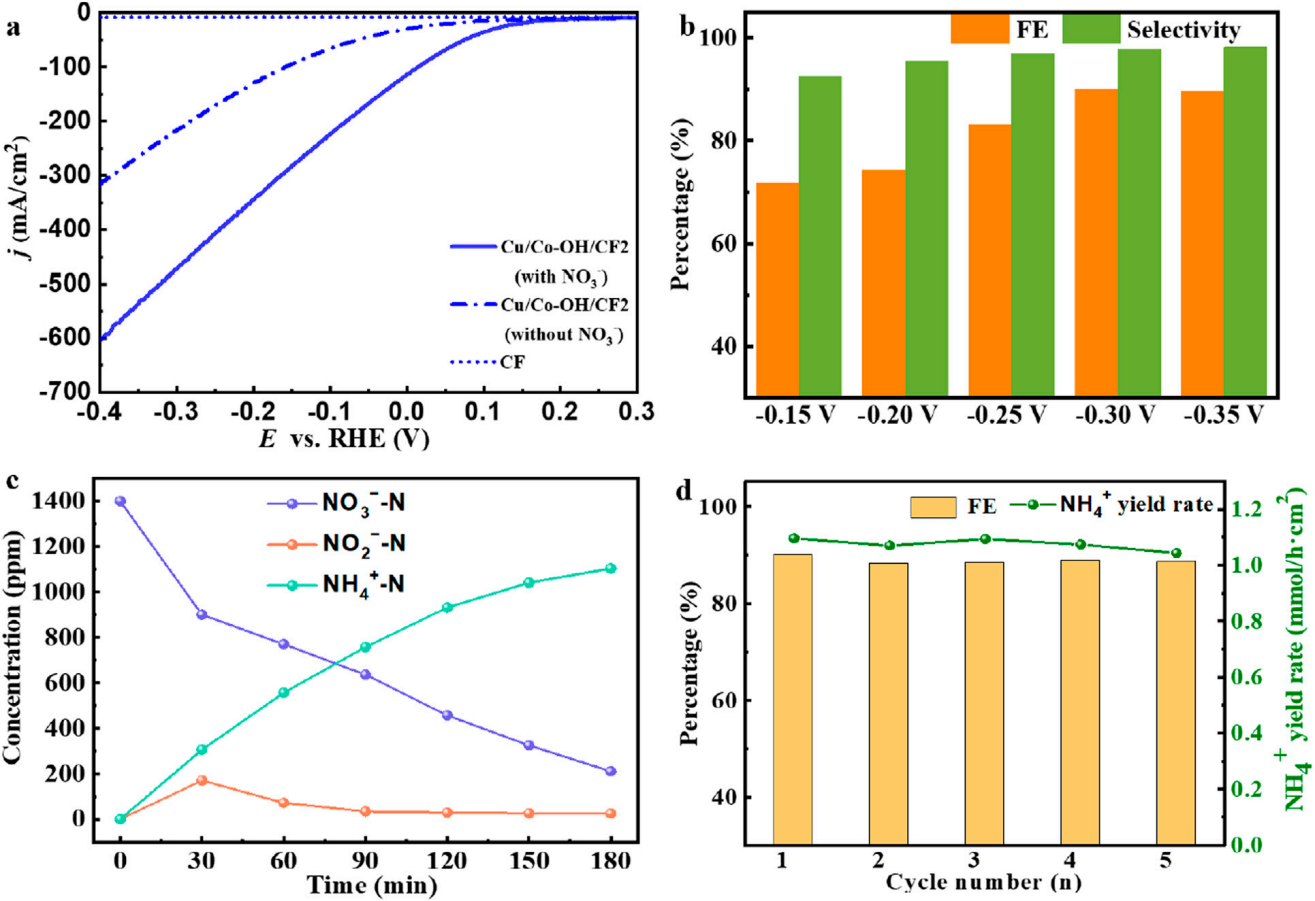
**(a)** LSV curve of Cu/Co-OH/CF2 in the presence or absence of NO_3_^−^ solution. **(b)** FE and selectivity of NO_3_^−^ reduction synthesis of ammonia by Cu/Co-OH/CF2 at different potentials. **(c)** The change process of each product during the reduction process. **(d)** Stability of 5 consecutive cyclic reactions.

The electrochemical measurements were conducted using the aforementioned three-electrode system, and the linear scanning voltammetry (LSV) curves depicting the dependence of current density were presented in Figure 5a. Remarkably, in the presence of NO_3_^−^, the current density exhibits a significant increase, indicating the effective catalytic activity of Cu/Co-OH/CF2 towards nitrate reduction. The CF substrate possesses negligible catalytic properties (Figure 5a). To further evaluate the electrocatalytic performance, the nitrate reduction performance of Cu/Co-OH/CF2 was investigated at different potentials (−0.15, −0.20, −0.25, −0.30, and −0.35 V vs. RHE), as shown in Figure 5b.

The results reveal that the faradaic efficiency (FE) steadily increases as the applied potential becomes more negative, reaching its maximum value of 90.06% at −0.30 V. Subsequently, at −0.35 V, FE slightly decreases to 89.68% due to the influence of competitive hydrogen evolution reaction (HER). Notably, at −0.35 V, the selectivity for the desired product NH_3_ is approximately 98.18%, indicating that only a negligible amount of NO_2_^−^ is generated. The time-dependent product content profiles further demonstrate the effectiveness of Cu/Co-OH/CF2 in the reduction of nitrate to ammonium (NH_4_^+^), as illustrated in Figure 5c. During the reaction, the concentration of nitrate continuously decreases while the concentration of ammonium steadily increases, confirming the successful conversion of nitrate to ammonium. Additionally, the content of nitrite (NO_2_^−^) exhibits a parabolic trend, peaking around 30 min of reaction time, after which it starts to decrease. This behavior indicates that Cu/Co-OH/CF2 exhibits remarkable catalytic activity for both nitrate and nitrite reduction.

To assess the stability of Cu/Co-OH/CF2, stability testing was performed over five cycles, each lasting for 3 h. The results, presented in Figure 5d, demonstrate that there is no significant decrease in the faradaic efficiency and NH_3_ yield, confirming the excellent stability of the electrocatalyst. Notably, in the first cycle, the NH_3_ yield reaches its maximum value of 1.096 mmol·h^−1^·cm^−2^. These findings highlight Cu/Co-OH/CF2 as an efficient and stable electrocatalyst for the reduction of NO_3_^−^ to ammonium (NH_4_^+^).

**FIGURE 6:**
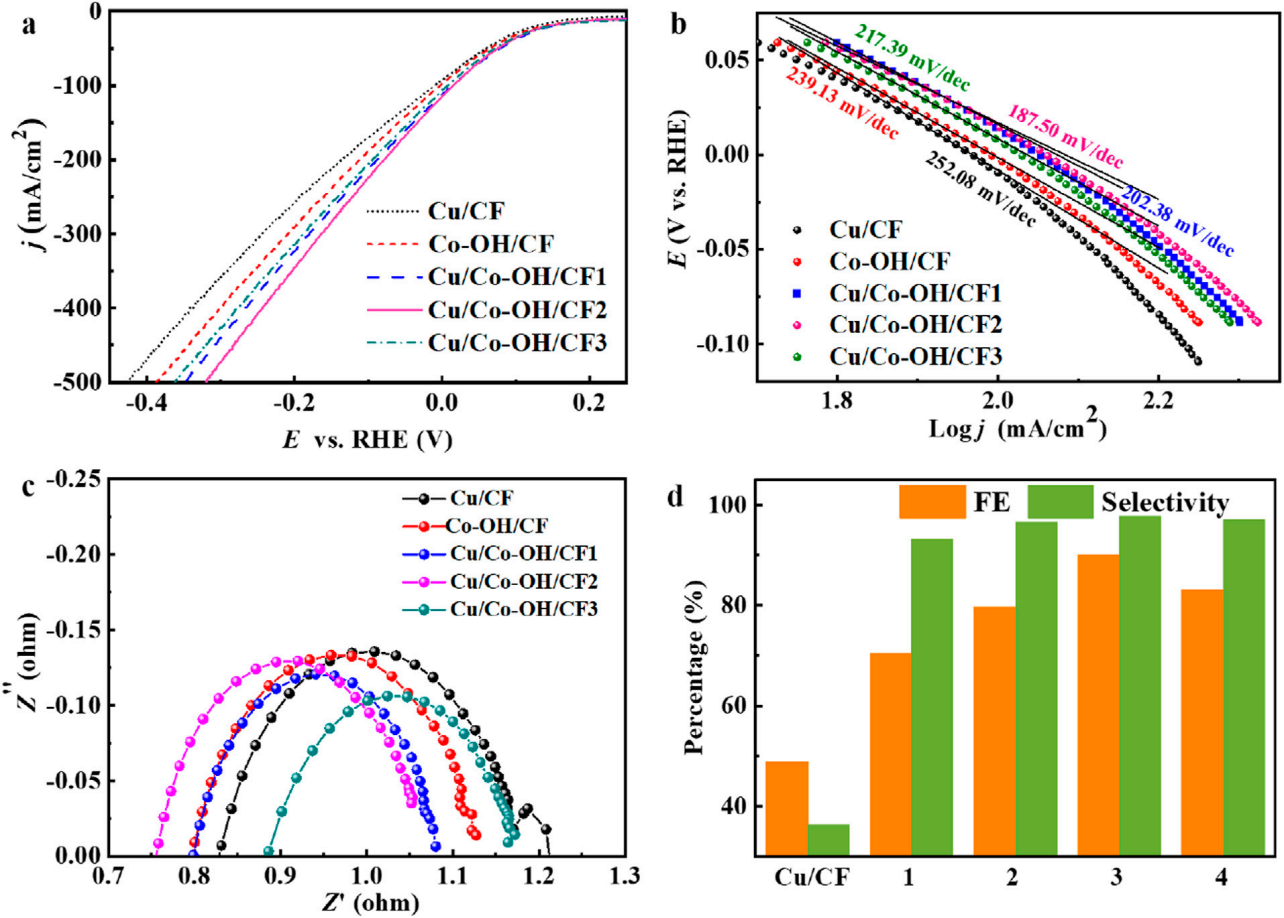
**(a)** LSV curves, **(b)** Tafel slopes, **(c)** impedance and FE and selectivity at −0.30 V of all samples. **(d)** FE and selectivity of NO_3_^−^ reduction synthesis of ammonia by different samples. Note in **(d)** 1 ↔ Co-OH/CF, 2 ↔ Cu/Co-OH/CF1, 3 ↔ Cu/Co-OH/CF2, and 4 ↔ Cu/Co-OH/CF3.

The electrocatalytic performance of Cu/Co-OH/CF2 was compared to other samples, including Cu/CF, Co-OH/CF, Cu/Co-OH/CF1, and Cu/Co-OH/CF3, in an electrolyte containing 1400 ppm NO_3_^−^-N. Linear sweep voltammetry (LSV) curves were employed to evaluate their performance, as depicted in Figure 6a. Remarkably, the current density of Cu/Co-OH/CF2 surpasses that of all other samples, indicating its superior electrocatalytic activity for NO_3_^−^ reduction. This suggests that the synergy between copper and cobalt in Cu/Co-OH/CF2 enhances the overall catalytic activity, making it more efficient in converting nitrate to ammonia.

The Tafel slope, a measure of the electrocatalytic kinetics, was analyzed to further assess the performance of the samples. As shown in Figure 6b, Cu/Co-OH/CF2 exhibits the lowest Tafel slope of 187.50 mV·dec^−1^. In comparison, the Tafel slopes of Cu/CF, Co-OH/CF, Cu/Co-OH/CF1, and Cu/Co-OH/CF3 are 252.08, 239.13, 202.38, and 217.39 mV·dec^−1^, respectively. These results indicate that Cu/Co-OH/CF2 possesses the fastest electrocatalytic kinetics for NO_3_^−^ reduction among the tested samples. The lower Tafel slope implies that the overpotential required for the nitrate reduction reaction is minimized, which is beneficial for improving the overall catalytic efficiency.

Impedance measurements were conducted to evaluate the electron transfer ability of the samples, as shown in Figure 6c. Cu/Co-OH/CF2 exhibits the smallest transfer resistance, confirming its rapid electron transfer capability. This result further supports the superior electrocatalytic performance of Cu/Co-OH/CF2. The reduced transfer resistance indicates that electrons can move more freely across the catalyst surface, which is essential for efficient nitrate reduction.

The faradaic efficiency (FE) of Cu/Co-OH/CF2 was determined at −0.30 V and compared to the FE of Cu/CF, Co-OH/CF, Cu/Co-OH/CF1, and Cu/Co-OH/CF3 under the same conditions (Figure 6d). Remarkably, Cu/Co-OH/CF2 achieves an FE of 90.06%, which is significantly higher than the FE values of Cu/CF (48.92%), Co-OH/CF (70.41%), Cu/Co-OH/CF1 (79.74%), and Cu/Co-OH/CF3 (83.21%). This result highlights the superior performance of Cu/Co-OH/CF2 in terms of nitrate reduction efficiency. The high faradaic efficiency indicates that a larger proportion of the applied electrical charge is effectively utilized for the nitrate reduction process rather than competing side reactions.

Regarding the selectivity for NH_3_ reduction, Cu/Co-OH/CF2 demonstrates the highest selectivity of 97.81%, approximately 2.7 times higher than that of Cu/CF (36.30%). This exceptional selectivity further emphasizes the superiority of Cu/Co-OH/CF2 as an electrocatalyst for nitrate reduction (Table 1). The high selectivity towards ammonia production is crucial for practical applications, as it minimizes the formation of unwanted byproducts, such as nitrite, and ensures a cleaner and more efficient ammonia synthesis process.

**TABLE 1:** Comparison of nitrate-N reduction with other cathodes.

Cathode	Electrolyte	Performance	Yield rate of NH3	Refs
Pd-Cu/SS	Pt wire | 0.01 M NaClO_4_, 0.6 mM NaNO_3_ (−0.2 V vs. SCE)	SE (NH_3_): 6%	—	Yao et al. (2019)
Zero-valent titanium (ZVT)	ZVT sheet | 25.9 mg N/L + 12 mg/L Cl^−^ (2.0 mA cm^−2^)	SE (NH_3_): 6%	—	Yao et al. (2019)
Ni-Fe0@Fe_3_O_4_	Pt sheet | 50 ppm NO_3_^−^ NaCl (5 mA cm^−2^)	SE (NH_3_): 10.4%	—	Jonoush et al. (2020)
Blended Sn_0.8_Pd_0.2_/SS	Graphite electrode | 0.008 M NaNO_3_ + 0.1 M HClO_4_ DC current = −0.04 A	SE (NH_3_): 14%	—	Yao et al. (2019)
Pd-Cu/γAl_2_O_3_	Graphite plate | 50 ppm NO^3−^-N (10 mA cm^−2^)	SE (NH_3_): 19.6%	—	Zhang et al. (2016)
Cu/rGO/graphite plat	Ti/RuIrO_2_ | 0.02 M NaNO_3_ + 0.02 M NaCl (−1.4 V)	SE (NH_3_): 29.93%	—	Rahimi et al. (2018)
Co_3_O_4_/Ti	Ir-Ru/Ti | 0.05 M Na_2_SO_4_, 50 mg L^−1^ NaNO_3_ (10 mA cm^−2^)	SE (NH_3_): 32%	—	Jonoush et al. (2020)
Co_3_O_4_-TiO_2_^−^PVP	IrO_2_-RuO_2_/Ti | 0.1 M Na_2_SO_4_, NaNO_3_ (10 mA cm^−2^)	SE (NH_3_): 73%	—	Su et al. (2016)
Fe@N-C	Platinum mesh | 50 mM Na_2_SO_4_ + 50 ppm NO_3_^−^ (−1.3 V)	SE (NH_3_): <75%	—	Cheng et al. (2019)
NFP	NFP/Pt | 0.5 M Na_2_SO_4_, 80 mg L^−1^ NaNO_3_ (−1.2 V vs. Hg/HgO)	SE (NH_3_) 89.1%; FE (NH_3_): 99.2%	0.056 mmol h^−1^ mg^−1^	Ya et al. (2021)
TiO_2-*x*_	Pt plate | 0.5 M Na_2_SO_4_, 50 ppm NO_3_^−^-N (−1.6 V vs. SCE)	SE (NH_3_): 87.1%; FE (NH_3_): 95.2%	0.045 mmol h^−1^ mg^−1^	Jia et al. (2020)
Co-NFs/CP	Pt-wire: 0.1 M Na_2_SO_4_ + 500 ppm NO_3_^−^ (−0.9 V vs. RHE)	FE (NH_3_): 91.15%	2865 μg·cm^−2^·h^−1^	Jiang et al. (2024)
1-Cu NPs@NDC	Pt-wire | 0.1 M Na_2_SO_4_ + 0.01 M NO_3_^−^ (−0.9 V vs. RHE)	FE (NH_3_): 99.85%	0.485 mmol mg^−1^ h^−1^	Chen et al. (2025)
Cu/Co-OH/CF2	Pt mesh | 0.1 M KOH and 0.1 M KNO_3_	SE (NH_3_): 97.81%; FE (NH_3_): 90.06%	1.096 mmol·h^−1^·cm^−2^	This work

The efficiency and selectivity of nitrate reduction were evaluated for different electrocatalysts in this study. The initial sample, Cu/CF, exhibited the lowest nitrate reduction efficiency and the poorest selectivity for the final product. The introduction of Co, as observed in the Co-OH/CF sample, resulted in a coverage of the CF substrate (as shown in Figure 3b), hindering effective contact between CF and the nitrate solution. Although the suppression of the competing hydrogen evolution reaction (HER) improved the nitrate reduction efficiency to some extent, the enhancement was not significant. However, the selectivity improved substantially due to the presence of Co(OH)_2_.

In the case of samples Cu/Co-OH/CF1-3, the simultaneous introduction of Cu (in nanowire shape) and Co(OH)_2_ (in nanoplate shape) allowed for effective contact between the catalysts and the solution, leading to increased nitrate reduction efficiency and selectivity. However, as the content of Co(OH)_2_ increased, the conductivity of the working electrode decreased due to its poor conductivity, resulting in a decrease in nitrate reduction efficiency. Therefore, it can be concluded that the synergistic effect between Cu and Co(OH)_2_ plays a vital role in nitrate reduction and the selectivity of the final product. Co(OH)_2_ primarily contributes to selectivity by suppressing HER, while Cu (rather than CF) is responsible for nitrate reduction (Wang et al., 2025; Huang et al., 2025; Lv et al., 2025).

## 3 Materials and methods

### 3.1 Materials preparation

All chemicals were commercially purchased and used without further purification. Initially, a CF with dimensions of 5 × 2 cm^2^ and a thickness of 0.1 mm was selected. The CF had a pore size of approximately 300 μm, which provided a large surface area for the deposition of the catalyst. The CF was immersed in a solution consisting of 35 mL of deionized water, 0.45 g of urea, and 0.0125 g of copper (II) nitrate trihydrate [Cu(NO_3_)_2_·3H_2_O]. The resulting mixture was then transferred to an autoclave and heated to 120°C, maintaining this temperature for 12 h. After the reaction, the CF was thoroughly cleaned using sequential rinses with deionized water and ethanol through ultrasonic treatment. The resulting sample was designated as Cu/CF. The loading mass of the catalyst on the CF was approximately 53 mg.

To obtain a reference sample, the above procedure was repeated with the substitution of 0.0125 g of copper (II) nitrate trihydrate with 0.0125 g of cobalt (II) nitrate hexahydrate [Co(NO_3_)2·6H_2_O], while 0.15 g of ammonium fluoride (NH_4_F) was added to the reaction mixture. This reference sample was denoted as Co-OH/CF. The loading mass of the catalyst on the CF is approximately 59 mg.

Next, three additional samples were synthesized using the same method as the Co-OH/CF sample, with the only difference being the varying quantities of cobalt (II) nitrate hexahydrate. The amounts used were 0.0125 g, 0.025 g, and 0.05 g, resulting in the samples labeled as Cu/Co-OH/CF1, Cu/Co-OH/CF2, and Cu/Co-OH/CF3, respectively. The loading mass of the catalyst on the CF is approximately 59 mg, 65 mg, and 73 mg, respectively. It is important to note that the hydrothermal method for fabricating samples Cu/Co-OH/CF1-2 still employed a solution composed of 35 mL of deionized water, 0.15 g of ammonium fluoride, 0.45 g of urea, and 0.0125 g of copper (II) nitrate trihydrate.

### 3.2 Characterization of structures, morphologies and valence states

The crystal structures of all samples were analyzed using a Rigaku D/Max-2500 X-ray diffractometer with Cu K_α_ radiation (λ = 1.54056 Å). The range of 2θ values scanned spanned from 15° to 75°. Scanning electron microscopy (SEM) imaging was performed using an S-4800 instrument from Hitachi, Japan, to examine the morphologies of the samples. To analyze the valence states of Cu and Co, X-ray photoelectron spectroscopy (XPS) data were collected using an ESCALAB-250 instrument with an Al K_α_ radiation source. The hemispherical detector employed had an energy resolution of 0.1 eV, enabling precise characterization of the valence states of the elements within the samples.

### 3.3 NRR measurements

NRR measurements were conducted using an H-type electrolytic cell equipped with a three-electrode system to evaluate the catalytic performance of the samples. The electrolytic cell employed a Nafion-117 proton exchange membrane to separate the cathode and anode chambers. All electrochemical data collection was performed using the CHI-760E electrochemical workstation.

Prior to the NRR tests, each electrode of the sample was securely fastened with a metal clip to serve as the working electrode. During the specific experiments, 45 mL of an electrolyte solution comprising a mixture of 0.1 M KOH and 0.1 M KNO_3_ was added to both the cathode and anode chambers of the H-type electrolytic cell. A platinum mesh electrode was employed as the counter electrode, while an Ag/AgCl electrode served as the reference electrode. These three electrodes were assembled to form the three-electrode system necessary for the measurements.

After allowing for a reaction time of half an hour, a small volume of the reaction solution was extracted for subsequent analysis of the NH_4_^+^ concentration. This enabled the calculation of the nominal activity and Faraday efficiency (FE) of the NRR reaction. All experiments were conducted at room temperature, and the potentials were determined based on the Nernst equation, with the potential measured against the Ag/AgCl reference electrode converted to the potential of the reference reversible hydrogen electrode (RHE) using the equation *E*_*RHE*_ *= E*_*Ag/AgCl*_ + 0.0592 × PH + 0.2.

## 4 Conclusion

In this study, we presented a series of electrocatalysts synthesized on CF by adjusting the cobalt source content through a facile hydrothermal method. These catalysts were employed as working electrodes for efficient nitrate (NO_3_^−^) reduction, leading to the production of ammonia. Through experimental investigations, we established that Cu exists in a metallic state, whereas Co exists in an oxidized state within the catalyst composite. By carefully controlling the amounts of Co in the composite, we were able to achieve precise regulation of nitrate reduction efficiency and the selectivity of the final product. Importantly, we observed a remarkable synergistic effect between Co(OH)_2_ and Cu, which played a pivotal role in achieving high efficiency in nitrate reduction and ensuring desirable selectivity of the final product, namely ammonia. The results of this study provide valuable insights into the design and optimization of Cu/Co-OH/CF electrocatalysts for nitrate reduction. By tuning the Co source content, we can modulate the electrocatalytic performance, enabling enhanced faraday efficiency and selectivity. The understanding of the underlying mechanisms and the synergistic interplay between Co(OH)_2_ and Cu opens up new avenues for the development of advanced electrocatalysts for environmentally friendly nitrate reduction processes.

## Data Availability

The original contributions presented in the study are included in the article/supplementary material, further inquiries can be directed to the corresponding author.
